# Critical Appraisal of Drug Promotional Literature in Accordance With WHO Guidelines

**DOI:** 10.7759/cureus.27644

**Published:** 2022-08-03

**Authors:** Sonali B Rode, Harsh V Salankar, Nilesh T Katole, Anuradha T Deshkar, Amruta A Dadmal, Shailesh V Parate

**Affiliations:** 1 Department of Pharmacology, Jawaharlal Nehru Medical College, Datta Meghe Institute of Medical Sciences, Wardha, IND; 2 Department of Pharmacology, Datta Meghe Medical College, Datta Meghe Institute of Medical Sciences, Nagpur, IND; 3 Department of Clinical Research, School of Allied Health Sciences, Datta Meghe Institute of Medical Sciences, Wardha, IND; 4 Department of Forensic Medicine, All India Institute of Medical Sciences, Rishikesh, Rishikesh, IND

**Keywords:** safety information, brand name, who criteria, printed material, drug promotional literature

## Abstract

Background

Drug promotional literature (DPL) is used as a marketing tactic to publicize the introduction of new medications. As drug companies are promoting the literature for their brand products, bias is possible. Various studies have demonstrated that printed DPLs disseminated by pharmaceutical companies are typically skewed.

Material and method

A prospective, observational study was carried out in the outpatient departments of a tertiary care hospital to analyze the DPL of different pharmaceutical companies using WHO criteria for "Ethical criteria for medicinal drug promotion, 1988".

Results

Out of 192 DPLs analyzed, information regarding the generic name, brand name, amount of active ingredient, and manufacturer name was found in all the DPLs (100%). Though therapeutic uses were mentioned in 91% of DPLs, dosage schedule (regimen) was mentioned only in 60%. Drug safety information such as the side effects and significant adverse drug reactions, precautions and warnings, contraindications, and major drug interactions were present in 24%, 36%, and 20%, respectively. Address of the manufacturer and reference to scientific literature were present only in 63% and 53% of DPLs, respectively. References mainly were from journals, present in 71% of DPLs. Most of the claims made in DPLs were regarding efficacy (73%), followed by safety (34%).

Conclusion

In our study, not a single DPL fulfilled all the nine WHO criteria. A doctor should rigorously evaluate study findings before prescribing because misleading and incorrect information is now frequently found in this literature.

## Introduction

New drugs are introduced into the market daily in substantial numbers [1]. Medical professionals get a lot of information from drug houses in different forms. There is printed material in the form of pamphlets, booklets, and colorful displays suitable for the prescription table. Besides that, there is electronically mediated literature. Drug companies create literature for the use of the common person, which comes in the form of printed advertisements and electronic media [2]. Around one-third of all sales earnings are burnt out on promoting the products, which is twice the amount that is exhausted on research and development [3]. As drug companies are promoting the literature for their brand products, bias is possible. When coming across such literature, medical professionals should be aware of the quality of the literature. They should be sure of the correctness of the contents and lack of exaggerations of the claims. Drug promotional literature (DPL) distributed by the medical representative is an essential source of seeking information for busy medical practitioners. As drug companies are promoting the literature for their brand products, there is a possibility of partiality. Various studies have denoted that printed DPLs disseminated by pharmaceutical companies are usually biased [4,5]. In developing nations, access to unbiased pharmacological information is further complicated by a lack of time for reading medical literature [6]. Educating and raising awareness among the medical community about the negative effects of unethical medication advertising is crucial. The pharmaceutical industry's medication promotion activities are governed by the WHO criteria for "Ethical criteria for medicinal drug promotion, 1988" [7]. As per WHO, promotional claims should be true, reliable, informative, balanced, updated, and have authentic information [8]. At this juncture, it is worth considering the criteria WHO laid down for ethical medicinal drug promotional literature. Therefore, this study was carried out to analyze the DPL of different pharmaceutical companies using WHO guidelines.

## Materials and methods

It was a prospective, observational, single-centered study carried out in the outpatient departments of a tertiary care hospital. On February 15, 2022, the Institutional Ethics Committee of Datta Meghe Institute of Medical Sciences approved the study vide letter number DMIMS(DU)/IEC/2021-22/88. Later the study was conducted for three months, from March 2022 to May 2022. Printed DPLs marketing allopathic medications were gathered from the outpatient departments of medicine, pediatrics, skin, psychiatry, ophthalmology, obstetrics and gynecology, ENT, and orthopedics. DPLs promoting allopathic medications, orthopedic prostheses, medical devices, equipment, drug reminders, drug monographs, and drug name lists were not included. According to the "WHO criteria," the following information should be mentioned in promotional literature [7]: 1. Names of the active ingredients used, either international non-proprietary names (INN) or approved generic names of the drugs; (2) Brand name; (3) Amount of active ingredient per dose; (4) Other ingredients which are known to cause problems, i.e., adjuvant; (5) Approved therapeutic uses; (6) Dosage form or dosage schedule; (7) Safety information, including side effects and major adverse drug reactions, precautions, contraindications, and warnings and major drug interactions; (8) Name and address of manufacturer or distributor; (9) References to scientific literature as appropriate. All the DPLs were evaluated accordingly; data were entered in a Microsoft Excel sheet and analyzed. In addition to this data, DPLs were examined for several kinds of stated claims. The sources of references cited to support assertions were examined, including the journal, website, books, data on file, etc. In order to find the references listed in the DPLs, an internet search was conducted.

## Results

**Figure 1:**
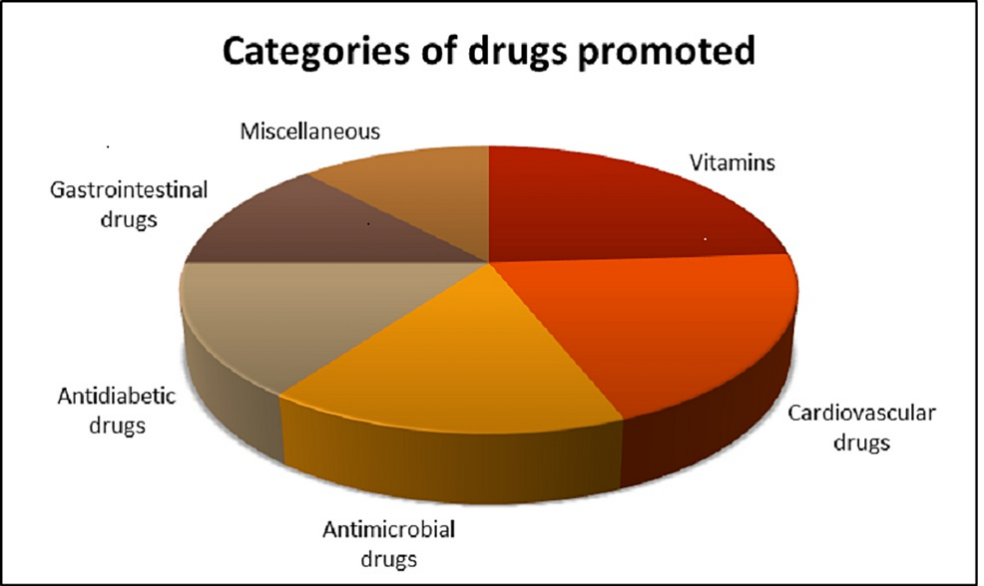
Promoted drug categories.

A total of 192 DPLs were analyzed for WHO criteria. Various categories of drugs promoted are shown in Figure 1. Vitamins (24%) were the most commonly promoted drugs, followed by cardiovascular drugs (20%), antimicrobials (16%), antidiabetic drugs (15%), GI drugs (13%), and miscellaneous drugs (12%), respectively. Out of 192 DPLs, 162 (84.4%) were single drugs, whereas 30 (15.6%) were fixed-dose combinations (FDC).

Analysis of DPLs as per the WHO guidelines is shown in Table 1. Information regarding the generic name of each active ingredient, brand name, amount of active ingredient, and manufacturer's name was found in all the DPLs (100%). Adjuvant was mentioned only in 25% of DPLs. Though therapeutic uses were mentioned in 91% of DPLs, dosage schedule (regimen) was mentioned only in 60%. Address of the manufacturer and reference to scientific literature were present only in 63% and 53% of DPLs, respectively.

**Table 1:** Analysis of DPLs in using WHO criteria. DPL: Drug promotional literature; INN: International nonproprietary name.

Sr. No.	WHO Criteria	No. of DPLs	Percentage
1	INN or approved generic name	192	100
2	Brand name	192	100
3	Amount of active ingredient per dose	192	100
4	Adjuvant	48	25
5	Approved therapeutic use	174	90.70
6a	Dosage form	186	96.90
6b	Dosage schedule	114	60
7a	Side effects and major adverse drug reactions	46	24
7b	Precautions and warnings	69	35.94
7c	Contraindications	38	19.80
7d	Major drug interactions	38	19.80
8a	Name of the manufacturer	192	100
8b	Address of the manufacturer	126	65.63
9	Reference to scientific literature	136	70.83

**Figure 2:**
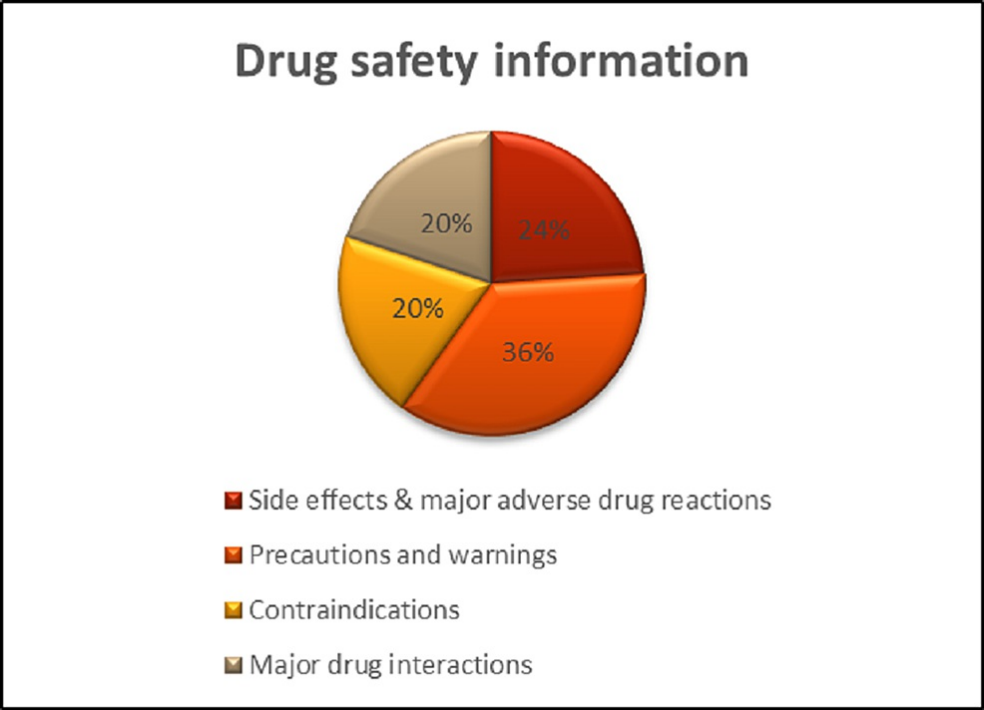
Drug safety information.

Side effects and significant adverse drug reactions were mentioned in 24%, precautions and warnings in 36%, contraindications in 20%, and major drug interactions in 20%, as shown in Figure 2.

**Figure 3:**
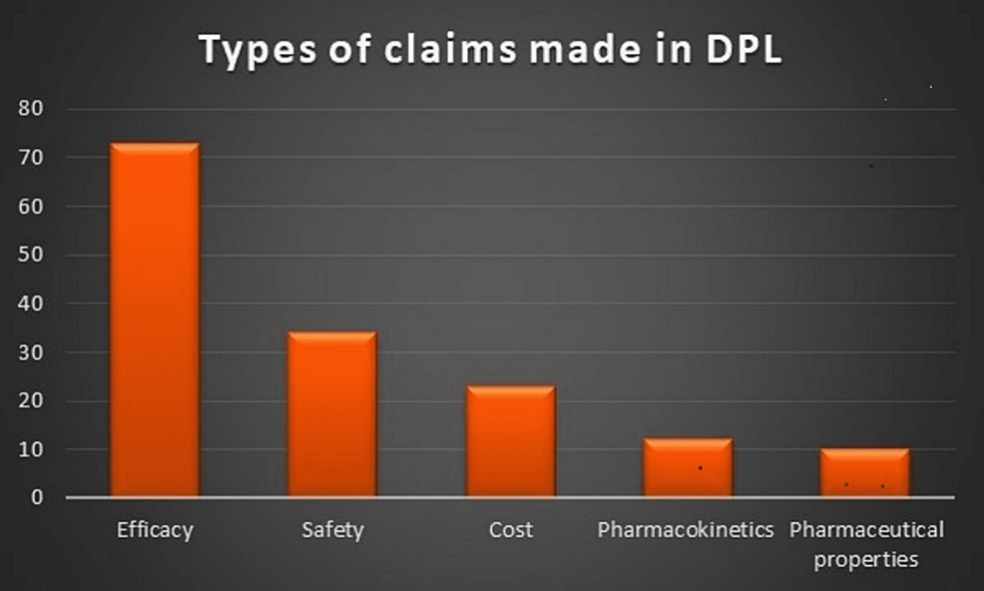
Various claims in DPLs. DPL: Drug promotional literature.

Sources of various references are shown in Table 2. References were cited in 136 (71%) DPLs, and about 70% of references were from journals, followed by websites 15%, books 11%, and other sources 5.15%. The majority of claims made in DPLs were regarding efficacy (73%), followed by safety (34%), cost (23%), pharmacokinetics (12%), and pharmaceutical properties (10%), as depicted in Figure 3.

**Table 2:** Various sources of drug information. DPL: Drug promotional literature.

Sr. No.	References	Number of DPLs (n = 136)	Percentage
1	References cited	136	70.83
2	Journal	94	69.11
	Before 2017	72	76.6
	After 2017	22	23.4
3	Website	20	14.71
4	Book	15	11.03
5	Other sources	7	5.15

## Discussion

DPLs are used as a marketing strategy to publicize the introduction of new medications. Drug manufacturing companies usually claim that their new products are much better when compared to current efficient and affordable goods, with which both physicians and consumers are familiar. Unfortunately, these materials are often misleading and confusing. Therefore, physician-targeted promotion through medical representatives is one of the most common tactics for drug promotion [2].

Vitamins (24%), followed by cardiovascular drugs, antidiabetic drugs, and antimicrobials, were the most commonly promoted drugs showing that pharmaceutical industries target prevalent conditions in the population. This finding was similar to the results of other studies [9-11]. Vitamins are the largest selling segment of the dietary supplement market. They are often purchased over the counter, without any specific indication. Vitamins should be prescribed for specific indications, and their indiscriminate use for preventing chronic diseases or general health promotion must be discouraged.

Brand name, generic name, the active ingredient, and dosage form were mentioned in 100% of the literature, consistent with a study conducted in Iraq and India [12,13]. We could make out that most of the DPLs quoted dosage schedules and therapeutic indications, but essential details regard­ing adverse drug reactions, contraindications, or drug inter­actions were missing. About 72% of the brochure did not mention the safety feature of the drug promoted. Similar findings were observed in other studies [10].

Information about adverse drug responses, medication interactions, precautions, and overdosage was the most overlooked part of drug marketing. These findings, which are consistent with other studies carried out in other parts of India, found that fewer than 10% of the literature mentioned adverse drug reactions [14,15]. This suggests that unethical drug promotion is widespread, which needs concern from all health authorities.

In this study, unsubstantiated claims about efficacy and safety were found in the brochures, which is consistent with similar studies [15,16]. Promotional brochures were full of unsubstantiated claims regarding safety and efficacy, and those claims were also therapeutically irrelevant. This aspect of the drug promo­tion was also highlighted in other similar studies [13,17].

The most important aspect of promotional literature is to look for sources of references that provide comprehensive updates to the physician from leading medical, national, or international journals. In our study, the references stating scientific information were mentioned in 71% of the brochures. In addition, up-to-date references (latest within the last five years) were stated in a few DPLs (23%). This is crucial for updating the clinicians so as to enhance their existing knowledge and practice of evidence-based medicine so this aspect should be looked after by the pharmaceutical companies.

During the review of various other studies of a similar type, we got the impression that well-known pharmaceutical companies do adhere to the WHO guidelines vigilantly. In contrast, the new and small setup pharmaceutical companies highlight only the positive points in their promotional literature. In our study, no single brochure contained complete information according to the WHO criteria. As misleading and wrong information is not uncommon in this literature, physicians should be made aware of the limitations of the current methods of medical industry promotions and the influence of marketing on prescribing behavior. The education of prescribers, the enforcement of current regulations, the creation of guidelines, and their application by pharmaceutical corporations for medication promotion are some corrective methods for this problem. 

The study's limitation is that it only assesses one sort of pharmaceutical company's promotional activities, namely printed promotional literature. However, gaining accurate and ethical information from promotional literature will be made possible by interventional research to gauge physicians' knowledge of these facts and inform them of the same.

## Conclusions

Evaluation of 192 printed promotional literature was done according to WHO's drug promotional criteria, but not a single prescription fulfilled all the criteria. While using drugs, especially new drugs on patients, a prescriber should analyze research findings attentively and draw conclusions, as misleading and incorrect information is now frequently found in promotional literature. This could lead to patients suffering unnecessary adverse effects and being prescribed more expensive products when cheaper alternatives are available. Therefore, the apex regulatory authorities controlling the drug industry in their respective countries should put effort into enforcing a law that will improve the quality of DPLs.
